# Supplementary figures and images for: A 12 kb multi-allelic copy number variation encompassing a GC gene enhancer is associated with mastitis resistance in dairy cattle
Source: PLoS Genet. 2021 Jul 21;17(7):e1009331. doi: 10.1371/journal.pgen.1009331 (PMC8328317; doi:10.1371/journal.pgen.1009331)

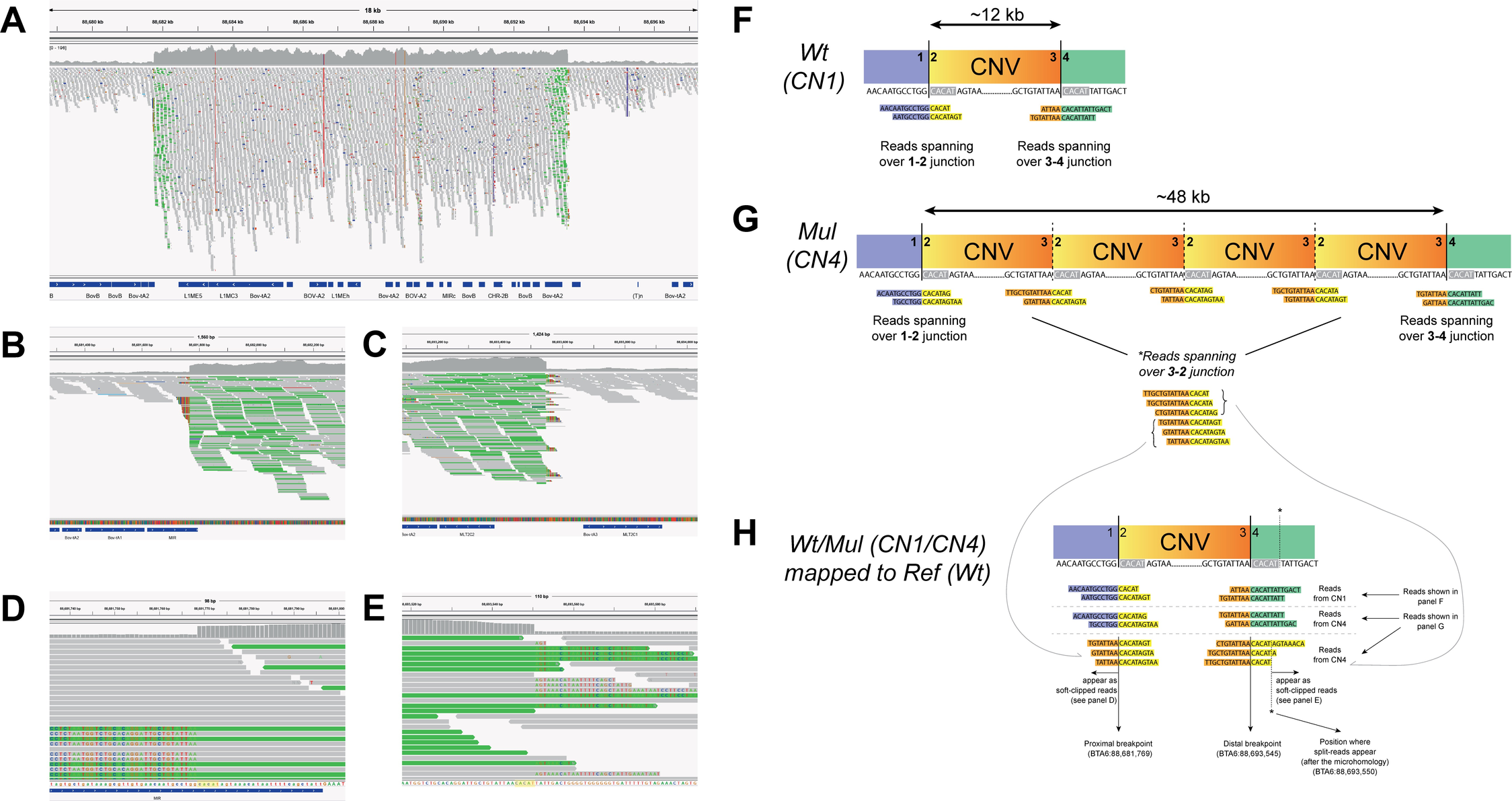

Supplement: S1 Fig — (A) The GC CNV (BTA 6:88,681,767–88,693,545) is shown in the IGV screen shot. The grey reads are normally mapped reads, whereas green ones are discordantly mapped reads, providing evidence for a tandem duplication. The sequencing coverage of the CNV region is higher than non-CNV region. (B) The left breakpoint is flanked by MIR repeat. (C) The right breakpoint does not overlap with repeats. (D, E) The proximal and distal breakpoints are zoomed in and the soft-clipped reads (positions where nucleotide sequences are written) information revealed the 5-bp microhomology “CACAT” at the breakpoints (marked as yellow). (F-H) A schematic overview demonstrating a tandem configuration of the GC CNV. (F) The start and end of the GC CNV (shown in yellow-to-orange gradient colour) is flanked by non-CNV background, forming two junctions shown as 1–2 and 3–4 (marked with solid vertical lines). Sequencing reads from Wt (CN1) haploid genome can uniquely aligned to the 1–2 junction (blue-yellow reads) and the 3–4 junction (orange-green reads). The microhomology sequence is marked with grey shade. (G) A haploid CN4 genome forms unique junctions spanning over 3–2 formed by tandemly aligned 12-kb segments (marked with dotted vertical lines). Thus, reads from haploid CN4 would have reads spanning over 3–2 junction (orange-yellow reads), in addition to reads aligned to junctions 1–2 and 3–4. (H) Alignment of a heterozygous genome (CN1/CN4) to the reference genome (Wt) is shown. Both CN1 and CN4 have reads spanning over 1–2 and 3–4 junctions that are present in the reference genomes; these reads can be uniquely mapped. Reads spanning over 3–2 junctions cannot be uniquely mapped to the reference genome; these reads will be discordantly mapped either at 1–2 or 3–4 junctions (grey lines). The 3–2 junctions reads can be aligned to the junction 1–2, however orange reads will appear as soft-clipped. Likewise, these reads can be aligned to the 3–4 junction. However, the 4 junction started with th [file pgen.1009331.s001.tif]

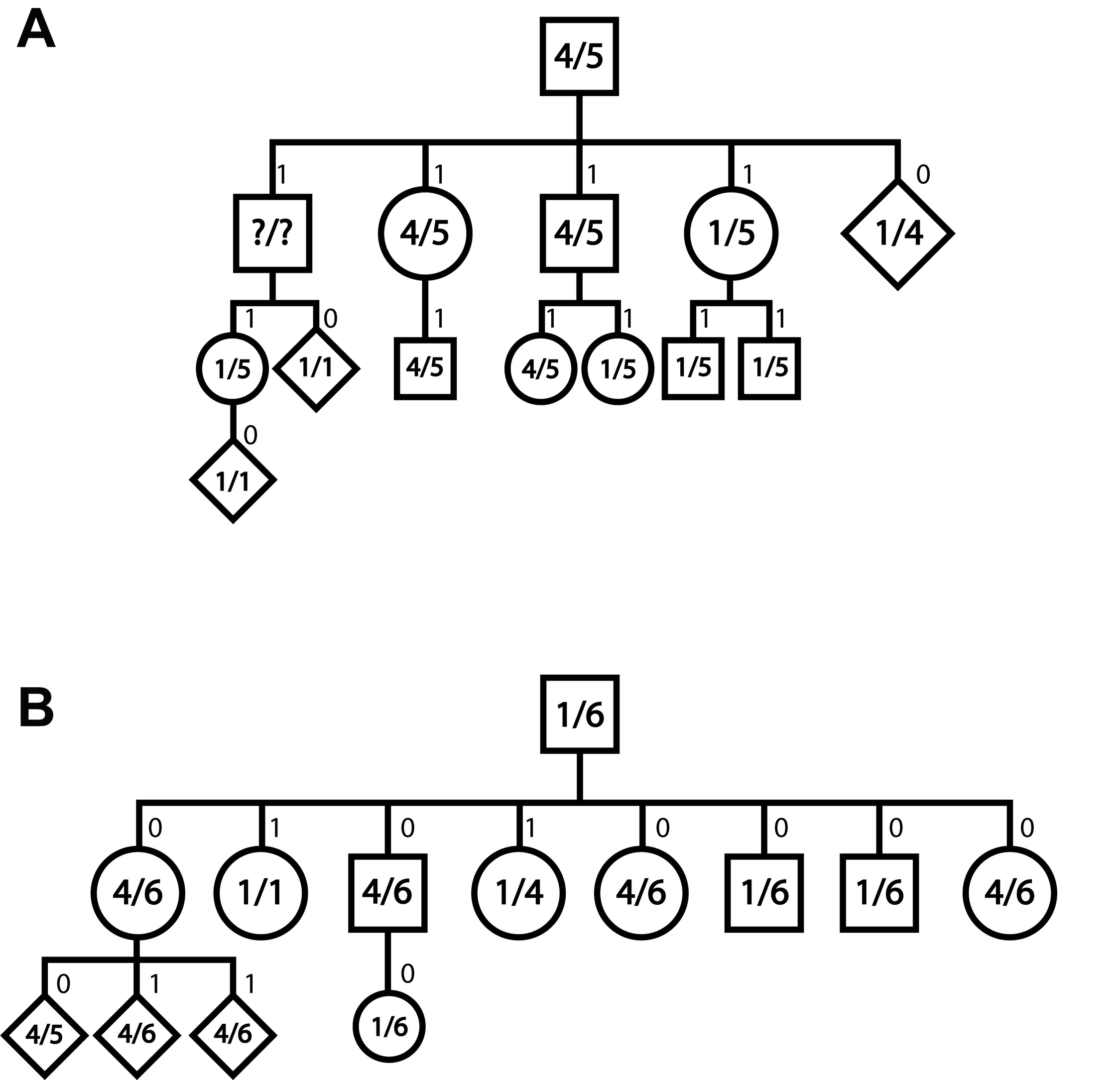

Supplement: S2 Fig — In the panel of 266 animals, we observed CN 5 and CN6 alleles are mostly segregating among a small number of related animals. To show that CN5 and CN6 alleles are truly segregating, transmission probabilities at each marker position were calculated with LINKPHASE3. Transmission probabilities were estimated for paternal haplotypes (1 and 0 indicated transmission of the paternal and maternal allele, respectively). (A) Largest family of CN5 carriers. Each circle indicates one individual and the number inside the circle indicates the GC CNV copy number genotype. The numbers above each individual stand for the transmission probability. Question marks mean individuals with no copy number information. Male animals are shown as squares, female animals are shown as circles, and animals with unknown gender are marked with diamonds. (B) Largest family of CN6 carriers. The legends are identical to panel (A). (TIF) [file pgen.1009331.s002.tif]

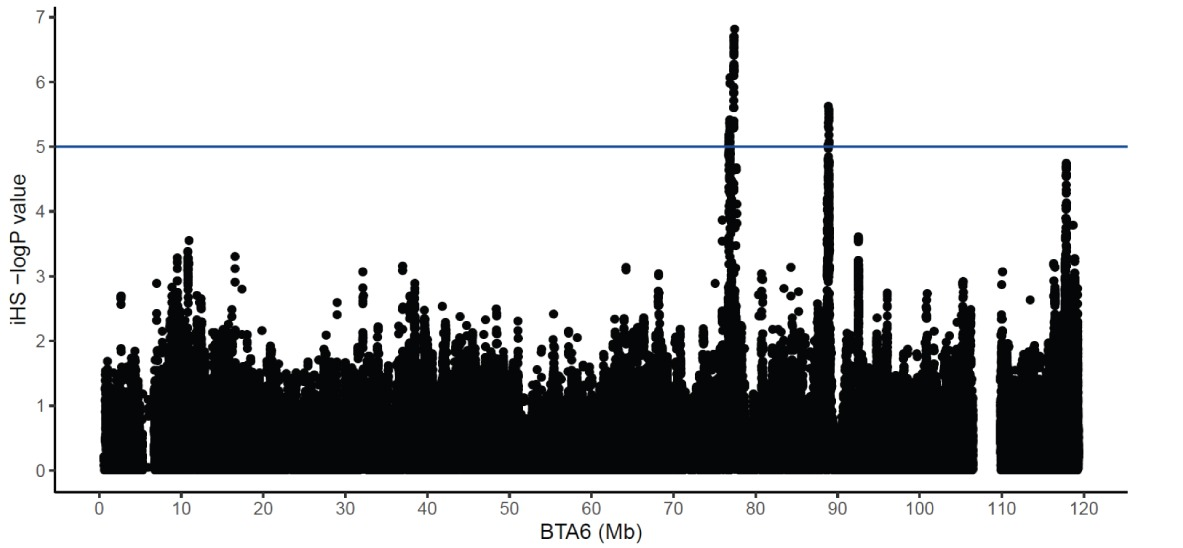

Supplement: S3 Fig — Chromosome-wide scan of integrated Haplotype Score revealed two iHS peaks with high significance (-log10P > 5), near BTA6:78 Mb and BTA6:89 Mb. (TIF) [file pgen.1009331.s003.tif]

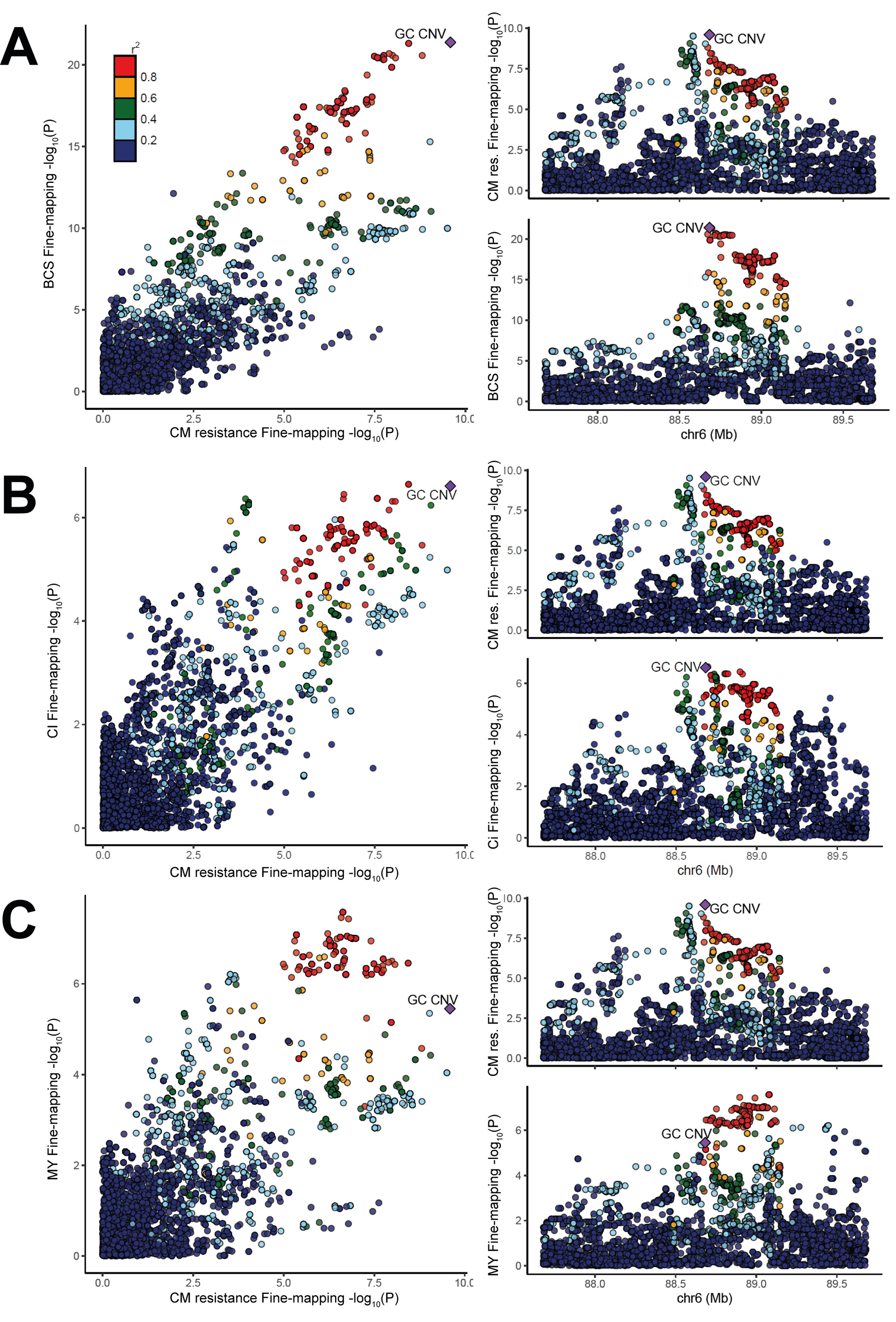

Supplement: S4 Fig — Colocalization of fine-mapping p-values of a pair of traits are showing that body condition score (BCS), calving interval (CI), and milk yield (MY) are having association signal near or at the CM resistance QTL region on BTA 6. (A) Colocalization between body condition score and CM resistance is shown in the left panel, together with the separate Manhattan plots for body condition score and CM resistance on the right. (B) Colocalization between calving interval and CM resistance. (C) Colocalization between milk yield and CM resistance. Panel layout of (B) and (C) is the same as panel (A). In each panel, the colours of dots indicate degree of LD (r2) with GC CNV (colour scale shown in the left upper corner of panel A). The purple diamond marks GC CNV. The figure was made with LocusCompare programme [99]. (TIF) [file pgen.1009331.s004.tif]

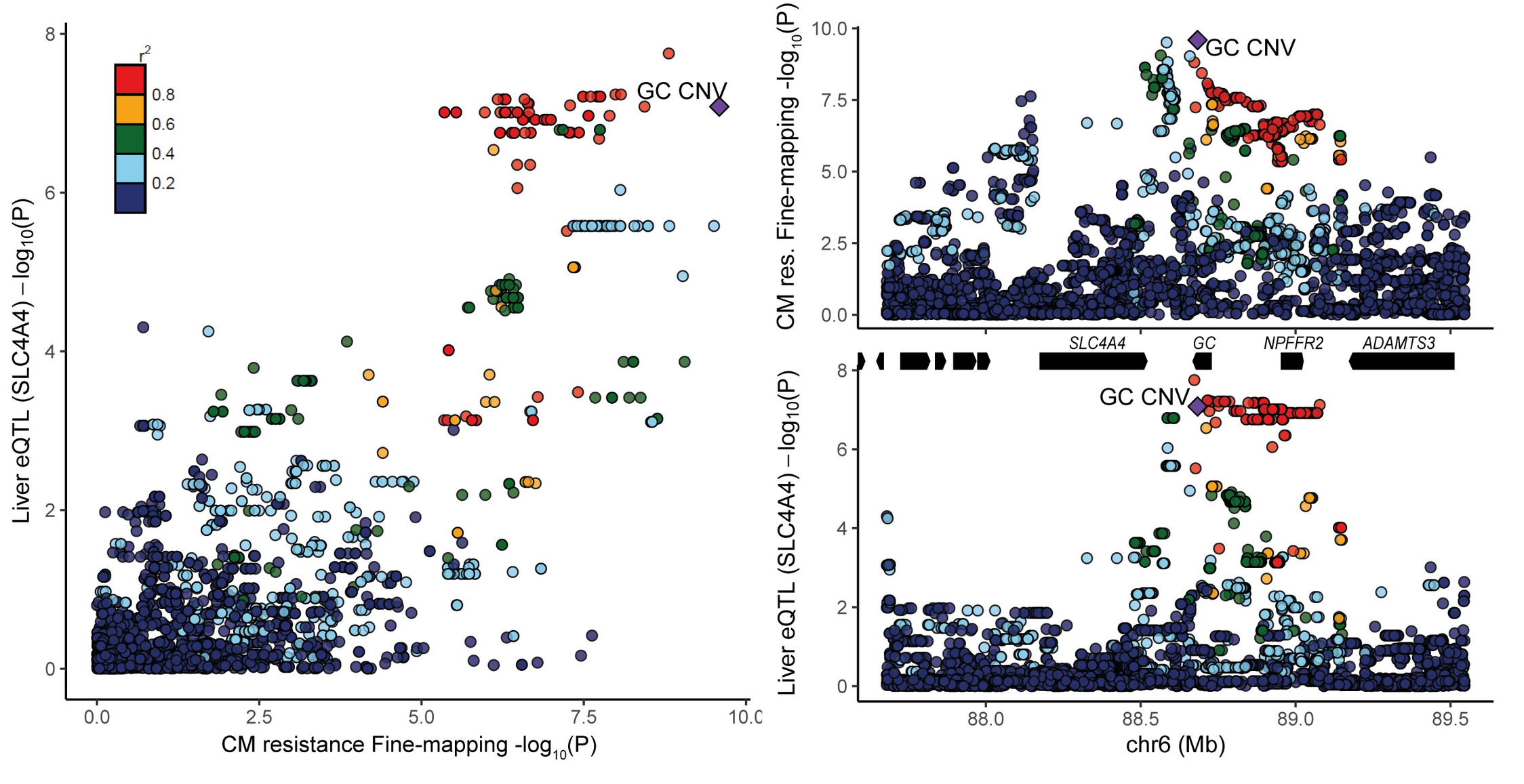

Supplement: S5 Fig — A colocalization plot for CM resistance fine-mapping and SLC4A4 eQTL mapping results is shown on the left side. The right upper panel is the CM resistance fine-mapping results and the right lower panel is the SLC4A4 eQTL mapping results. In between these two right panels are the genes located in this region. Six genes on the left part are AMBN, JCHAIN, RUFY3, GRSF1, MOB1B, and DCK. In each panel, the colours of dots indicate degree of LD (r2) with GC CNV (colour scale shown in the left upper corner of the colocalization figure. The purple diamond marks GC CNV. (TIF) [file pgen.1009331.s005.tif]
