# Supplementary material for: A 12 kb multi-allelic copy number variation encompassing a GC gene enhancer is associated with mastitis resistance in dairy cattle
Source: PLoS Genet. 2021 Jul 21;17(7):e1009331. doi: 10.1371/journal.pgen.1009331 (PMC8328317; doi:10.1371/journal.pgen.1009331)
Supplement: Attachment — Submitted filename: Response_to_editors_210325.docx [file pgen.1009331.s016.docx]

Response to the editors’ request:

Regarding the current manuscript, there is a consensus that the major accomplishment and potential impact of the work relates to the identification of the structural variant that affects GC activity. The path proposed in your email is generally acceptable:

(1) removing the genome-wide GWAS plot from the supplementary material;

(2) providing summary statistics for all of BTA6 as part of supplementary material or in a public database;

(3) revising the data availability statement to indicate that individual-level data is available without preconditions (e.g. authorship, collaboration) for academic use via an MTA.

**AU:** Thank you very much for considering our manuscript in your journal. We were asked to modify our manuscript as above to meet Plos Genetics data policy. We removed the genome-wide GWAS figure (previously S1 Fig), and instead added supplementary table of BTA 6 association mapping summary statistics (S1 Table). Furthermore, the data availability statement is revised, stating that the data will make available upon request based on a material transfer agreement. Lastly, part of manuscript (text and figures) referring to GWAS has been revised to association mapping and marked with highlight.
