# Supplementary material for: A 12 kb multi-allelic copy number variation encompassing a GC gene enhancer is associated with mastitis resistance in dairy cattle
Source: PLoS Genet. 2021 Jul 21;17(7):e1009331. doi: 10.1371/journal.pgen.1009331 (PMC8328317; doi:10.1371/journal.pgen.1009331)
Supplement: Attachment — Submitted filename: 210531_Rebuttal.docx [file pgen.1009331.s017.docx]

**Comments from reviewer 1**

In this manuscript, Lee an colleagues describe the identification of a 12 kb multiallelic CNV associated with resistance to clinical mastitis and propose a GC gene enhancer in this CNV as the putatative causative mechanism. The authors exploited various data sets (genomic, transcriptomic, epigenomic) and conducted a thorough analysis using state-of-the-art methodolgy. Using mendelian randomaization and HEIDI to disentangle the two candidate genes e.g. is very convincing. On the other hand the authors clearly state the limits of their data sets that do not allow to differentiate between LD and pleiotropy in terms of the effect on milk yield vs. mastitis. My only criticism would be that the manuscript could be written a bit more concisely (e.g. the part describing the CNV alleles).

**AU: We thank the reviewer for these positive comments on the manuscript. In response to the criticism of the length of the manuscript, we re-wrote association analysis and CNV allele part in a concise manner in line: 77-144.**

Minor issues:
- In line 111, you cannot use the term 'nomal', I think.

**AU: We re-wrote part of our manuscript following the reviewer’s suggestion, and now the sentence the reviewer addressed is removed from the manuscript.**

- Regarding the SLC4A4 gene: an eQTL for this genes was also found - what is known about the function of this gene? Any implications?

**AU: Solute Carrier Family 4 Member 4 gene (*SLC4A4*) is highly expressed in the kidney and pancreas. The known functions of *SLC4A4* include bicarbonate secretion regulation. We incorporated this information at line 208-209 as following:**

“Additionally, cis-eQTL for **Solute Carrier Family 4 Member 4 gene (*SLC4A4*), involved in bicarbonate secretion and associated with renal tubular acidosis,** was found in BTA 6:88.67-89.07 Mb (-log10P > 7, S5 Fig).”

- Effects for CI and BCS were found. These traits would be genetically correlated with MY, wouldn't they?

**AU: The negative genetic correlations between BCS and MY, and CI and MY have been reported. To make this point clear in our manuscript, we cited two studies: (i) The genetic relationship between calving interval, body condition score and linear type and management traits in registered Holsteins (Pryce *et al.,* 2000) and (ii) Genetic relationships among body condition score, body weight, milk yield, and fertility in dairy cows (Berry *et al.,* 2003). Now, the sentence in line 330-332 reads:**

“**Indeed, GC CNV showed strong associations with BCS and CI, which are known to be negatively correlated with MY (e.g. cows with low BCS have longer CI and higher MY [78,79]), indicating pleiotropy (S4 Fig).** “

**Comments from reviewer 2**

I thought was a very nice, well written paper. Proving a variant is causative is difficult without extensive lab work e.g. editing the functional site, but the authors make a strong case for the importance of the CNV. I have no major concerns about anything in the manuscript but there were some things that would be good if could be addressed.

**AU: We thank the reviewer for these positive comments on the manuscript.**

1 – I was a bit surprised the authors hadn’t tried to determine the actual sequence of the CNV. For example, in theory the extra copies of the sequence may not even come from this location i.e. they could be insertions somewhere else in the genome but because the extra copies aren’t in the reference genome all their reads map back to this location. The mendelian inheritance would suggest they are at least in the same region but at present just demonstrate more reads at this location than expected. Although one possibility is to Sanger across the breakpoints, as the CNV is relatively common there seems a reasonable chance one of the other current genome assemblies (Brahman, Angus etc) may have long reads/optical mapping data corresponding to the CNV. Or even the Hereford given could have been a heterozygote. Hadn’t seemed to check updated Hereford assembly and data. Understanding the sequence of the CNVs is likely important to understand its effects.

**AU: Thank you for your critical comments on the structural arrangement of the GC CNV. We have three-folds of evidence supporting a tandem arrangement of the GC CNV. Firstly, we reconstructed haplotypes in the 266 animals using family linkage information with LINKPHASE3 software. Linkage-based phasing allowed us to confirm that the GC CNV is segregating together with the surrounding haplotype on BTA6, which was elaborated in our haplotype analysis (line number: 129-134). Secondly, the LD with the CNV tagging SNPs (Figure 3B) and the SNPs outside of, but adjacent to the GC CNV was high, as demonstrated in Fig 4B and Fig 5C. We believe a transpositional duplication event would lead to low LD, as the duplicated segment would be inserted into a novel haplotypic background. Thirdly, a proof of transpositional event would be (discordant, split, or clipped) reads bridging the GC CNV and another genomic region where other copies exist. Manual inspection of the sequencing data using Integrative Genome Brower did not reveal reads supporting a transpositional event.**

**In an attempt to provide further evidence, we parsed out a read with partially unmatching sequences, located at the distal breakpoint (Figure S1 panel E). This read, aligned at chr6:88,693,494-88,693,550 of the reference genome, had CIGAR string of “57M47S”, indicating alignment of 57-bp sequence, whereas 47-bp soft-clipped sequence was not mapped contiguous to the 57-bp sequence. Note that the last 5-bp sequence of the 57-bp is the microhomology sequence “CACAT”. We aligned the contiguous stretch of 104-bp sequence (CCTTACCTGAGAACCATTACCTCTAATGGTCTGCACAGGATTGCTGTATTAACACATAGTAAACATAATTTTCAGCTATTGAAATAATCCTTCCTAATTGACCC) to UMD3.1 using UCSC genome browser blat tool. As expected, the 57-bp was mapped to chr6:88,693,494-88,693,550. The 47-bp soft-clipped sequence aligned to the starting part of the GC CNV (chr6:88,681,769-88,681,820). Notably, the UCSC blat tool returned the results of 52-bp alignment, instead of 47-bp, since the microhomology sequence “CACAT” that bridged 57-bp and 47-bp sequences are located at the start of the CNV of the original read was aligned as well.**

**Additionally, in search of animals that have breakpoints spanning over multiple copies, we inspected a recently published hybrid cattle genome (GCF_003369695.1) and a newer Hereford genome (ARS-UCD1.2). Alignment of the 104-bp sequence (mentioned above) resulted in alignment to two locations similar to the UMD3.1 results, hinting that the haploid genomes used for building the reference genomes do not have GC CNVs (for the hybrid cattle genome, 57-bp sequence aligned at chr6:86,534,684-86,534,443 and 52-bp sequence aligned at chr6:86,521,723-86,521,774; for ARS-UCD1.2, 57-bp sequence aligned at chr6:** **86,961,377-86,961,433 and 52-bp sequence aligned at chr6:86,949,653-86,949,704). Two unpublished long-read sequencing data was inspected (Two Belgian Blue animals), however they were not GC CNV carriers.**

**Lastly, we extended Figure S1 (adding panels F-H) to better explain the sequencing reads evidence supporting the tandem configuration of the GC CNV. In these panels, we showed that the GC CNV creates novel junctions bridging the CNVs in tandem that are not present in the reference genome. Hence the reads spanning over these novel junctions are discordantly mapped to start and end of the GC CNV, where some of these reads are either hard- or soft-clipped. The explanation for these panels are added in the legend at line number 925-937:**

**“(F-H) A schematic overview demonstrating a tandem configuration of the GC CNV. (F) The start and end of the GC CNV (shown in yellow-to-orange gradient colour) is flanked by non-CNV background, forming two junctions shown as 1-2 and 3-4 (marked with solid vertical lines). Sequencing reads from Wt (CN1) haploid genome can uniquely aligned to the 1-2 junction (blue-yellow reads) and the 3-4 junction (orange-green reads). The ‘CACAT’ microhomology sequence is marked with grey shade. (G) A haploid CN4 genome forms unique junctions spanning over 3-2 formed by tandemly aligned 12-kb segments (marked with dotted vertical lines). Thus, a haploid CN4 would have reads spanning over 3-2 junction (orange-yellow reads), in addition to reads aligned to junctions 1-2 and 3-4. (H) Alignment of a heterozygous genome (CN1/CN4) to the reference genome (Wt) is shown. Both CN1 and CN4 have reads spanning over 1-2 and 3-4 junctions that are present in the reference genome; these reads can be uniquely mapped. Reads spanning over 3-2 junctions cannot be uniquely mapped to the reference genome; these reads will be discordantly mapped either at 1-2 or 3-4 junctions (grey lines). The 3-2 junctions reads can be aligned to the junction 1-2, however orange reads will appear as soft-clipped. Likewise, these reads can be aligned to the 3-4 junction. However, the 4 junction started with the “CACAT” sequence. Hence, the sequences after the microhomology will appear as soft-clipped (marked with dotted line and asterisk).”**

2 – Along these lines I was a bit confused by Figure 3A. If the CTCCT, single copy haplotype was the ancestral state as the authors suggest I’m not quite sure how changed to multiple copies of TCTTG haplotype with no intermediate states being observed? Any hypotheses? Normally to infer ancestral state would use alignments to other breeds/species. E.g. what is the state of this region in the Brahman genome i.e. indicus breeds.

**AU: Thank you for your insightful comments. We inspected haplotypes sequence in species related to domestic cow (Bos indicus, wild yak, American bison, and goat) using Ensembl multi-species alignment database. The phylogeny tree obtained based on ~5-kb sequence (BTA6:88,683,517-88,688,920; spanning over five GC CNV tagging SNPs) was concordant with the species tree in a recent large-scale ruminant phylogeny study (Large-scale ruminant genome sequencing provides insights into their evolution and distinct traits, Chen et al 2019). The sequences of the five GC CNV tagging positions were:**

**Bos Taurus: CTCCT**

**Bos Indicus: CCTTT**

**Wild yak: CTCCT**

**American bison: CCTTT**

**Goat: CCTCT**

**Sheep: CCTCT**

**This information suggests: 1) the ancestral allele within Bovinae family based on parsimony theory is CCTTT (shown in American bison), 2) the fact that the genus Bos (B. Taurus and B. Indicus) carrying CCTTT and CTCCT implies either incomplete lineage sorting or gene flow.**

**In short, we do not consider CTCCT and TCTTG observed in our data set (B. Taurus) as the Bovinae ancestral haplotypes. Instead, CTCCT and TCTTG are both mixtures of ancestral and non-ancestral alleles. Of note, we suspect that the 5^th^ position (BTA6:88,688,920) is prone to frequent mutations given the local genomic context (GT repeat)**

**Furthermore, our haplotype analyses showed that the CNV carrying haplotypes share long IBD segments, indicating a recent common ancestral haplotype. The shared haplotype is associated with four, five, and six copies. This suggests that the copies appeared on that haplotype. All the other haplotypes in the population carry a single copy. The most parsimonious hypothesis is that originally all different haplotypes segregating in the population had a single copy, and that relatively recently (given the length of haplotype sharing) additional copies appeared on a single background.**

**Regarding the absence of the intermediate copies (e.g. CN2 or CN3), we currently do not have a standing hypothesis. Possible explanations could be: 1) the intermediate CNs existed before, however were lost due to drift, or 2) the intermediate CNs have not existed; instead, a single mutagenic event gave rise to the CN4 allele. A rolling circle amplification mechanism was proposed as a possible mechanism of a de novo high order amplification event in human (Complex Genomic Rearrangements at the PLP1 Locus Include Triplication and Quadruplication, Beck *et al.,* 2015). Along the line, we would like to remark on a trait-associated multiallelic CNV example reported in cattle, analogous to our GC CNV (A structural variant in the 5’-flanking region of the *TWIST2* gene affects melanocyte development in belted cattle, Mishra *et al*., 2017). The diploid CNs of this CNV range between 2 and 12, where most animals were either CN2 non-belted cattle or CN 5 (CN1/CN4) and CN 8 (CN4/CN4) belted cattle, with some few intermediate CN animals. This distribution suggests CN1 and CN4 as the major alleles for this CNV locus, like GC CNV.**

3 – The authors clump copy numbers 4-6 together and analyses the CNV as if biallelic. Obviously if could show expression levels/phenotypes depend on the precise number of copies would be stronger evidence that the CNV may be causal.

**AU: Thank you for your constructive suggestion. We fully agree that showing the altered expression level, depending on the discrete copy number states, would provide even more robust evidence for the causative effect of the GC CNV. In our understanding, this validation would require RNA extraction from liver samples obtained from a significant number of animals carrying CN5 or 6. We failed to meet these requirements in the current study given that : 1) CN5 and CN6 haplotypes were rare and only shown in related animals (allele frequency of 0.03 and 0.05, respectively in 266 WGS animals; see Figure 3A), and 2) currently we do not have liver tissues stored for expression analyses for animals carrying CN5 or 6.**

4 – Could impute expression values into wider GWAS cohort using a TWAS approach taking into account other cis-eQTLs to help differentiate between the two potentially regulated genes. These genes are unlikely to be solely regulated by just variants at this CNV region, so if the downstream phenotype is truly mediated by the expression level of the causal gene a more comprehensive TWAS approach incorporating the full set of cis-regulatory variants would potentially be informative.

**AU: Thank you for your insightful comments. Following the suggestion from the reviewer, we aimed to detect variant(s) affecting expression of *GC* and *SLC4A4*, additionally to the GC CNV. To achieve this, we mapped *cis*- and *trans*- eQTL and used GC CNV genotypes as a covariate in the model. For *GC*, we mapped eQTL for the canonical and alternative transcripts separately, as done in our manuscript. The strong eQTL signal near the GC CNV disappeared after including the covariate. Additionally, we discovered a weak eQTL signal near BTA6:88.84-88.88 for the canonical *GC* transcript (raw P-value = 3.05E-07, FDR = 4.37E-05). This region showed a putative enhancer signal (see Enhancer (3) in Figure 6A). Regarding to the *cis*-eQTL for the alternative *GC* transcript and *SLC4A4* and the *trans*-eQTL, we have not discovered significant signals (FDR < 0.001). Taking our original analyses and the additional analyses done here together, we deem that the GC CNV as the main *cis*-regulatory variant for *GC*, where there might be an additional cis-regulatory variant (enhancer (3)) playing a role. However, note that our data set (n=175) might be underpowered to detect weak to moderate eQTL signals and further investigation with a larger dataset might be appropriate to follow up on it.**

More minor points:

5 – I would probably have performed the eQTL analysis excluding any RNA-seq reads mapping to the CNV region i.e. just using the read counts across the other exons. As otherwise the results could be confounded.

**AU: The GC gene has two transcripts: a canonical form consisting of 13 exons that do not encompass the GC CNV and an alternative form that skips the 13^th^ exon and use an alternative exon (schematic overview shown in Fig 5A). In our eQTL mapping, we mapped eQTL for these two transcripts separately. The eQTL mapping results for the canonical transcript are shown in Fig 5 D, E, F, whereas ones for the alternative transcript are shown in Fig 5 G, H, I. These figures show that the GC CNV effect is significant when the reads mapped to the GC CNV are not counted. To make this point clear, we revised the manuscript at line 196-198 as following:**

“Our RNA-seq data detected two different GC transcript isoforms: a canonical and an alternative transcript (Fig 5A). The former consisted of 13 exons**, not encompassing the GC CNV,** and accounted for a majority of overall GC expression (~98%). The latter shared the first 12 exons with the canonical transcript, however, it used an alternative 3’ exon, the 14th exon, which is located inside the GC CNV.”

6 – Should be “did not have any evidence of a functional role”

**AU: We rephrased the sentence at line 101 as suggested.**

7 – “Bovine liver epigenomic data, interrogating two histone modifications (ChIP-seq for H3K27ac and H3K4me3 marks) [43] and open chromatin regions (ATAC-seq) [manuscript in preparation]” Would be good to know more details even if full details coming in another manuscript e.g. breeds studied etc. Can you infer the CNV state of the samples the chromatin came from by looking at the presence of the alleles in the reads that differentiate the CNV states? If discordance between their CNV number and the reference is mapping the chromatin data to the CNV complicated by the fact it is a CNV?

**AU: Thank you for your insightful comments. The breed information and the GC CNV genotypes of the animals used for generating functional data are as following:**

**(i) The information for bovine liver ChIP-seq data, detailed in Table S1 of Villar *et al.,* (2015), does not provide the breed information. Of the four liver ChIP-seq samples, we used one named “Bull_5” to visualize the ChIP-seq peaks in Figure 6. The H3K27Ac ChIP-seq reads mapped to the GC CNV tagging SNPs in this sample was T/C, C/T, T/C, T/C, G/T (sequencing coverage: 2-8X). Although this animal has genotypes associated with the GC CNV (TCTTG), we could not conclude that this animal has GC CNV given that: 1) the negative control file did not show coverage increase in the GC CNV region, and 2) these SNPs did not show a strong allelic imbalance.**

**(ii) The liver ATAC-seq data is obtained from a study under preparation (a provisional title: “Dissecting genetic contribution of variants residing in putative regulatory elements to improve accuracy of genomic prediction in bovine” by Haruko Takeda). The data was generated from a male Holstein Friesian calf, and the breed information is now added in the materials and methods section (line 484). Genomic DNA sequencing data of this calf showed increased read-depth in the CNV region, and the CNV-tagging SNP genotypes were: T/T, C/C, T/T, T/T, G/G (sequencing coverage: 2-8X). Based the SNP genotypes and the increased read-depth, we deem this animal is a homozygous carrier of the GC CNV.**

**The information elaborated above (genotypes and number of reads supporting each allele on the five GC CNV tagging SNP positions) is now provided as supplementary table 9. Also, to acknowledge the fact that we can infer the putative GC CNV genotype based on the SNP genotypes (if supported by allelic imbalance or WGS read-depth), we added the following sentence to the legend of the figure 6 at line 895-898:**

“**The GC CNV genotype of the ATAC-seq sample was Mul/Mul (inferred based on CNV tagging SNP genotypes and read-depth increase in WGS data). The GC CNV genotype of the ChIP-seq sample was unknown**.”

**Lastly, we used MACS2 software to perform peak calling for both ChIP-seq and ATAC-seq data. This software takes genome background file as an input and correct for the background level (e.g. increase of read-depth due to CNVs) in calling peaks. Thus, we consider the ATAC signal shown in the male HF calf is not merely driven by the GC CNV.**

8 – “The GC CNV and the SLC4A4 eQTL lead SNP were in high LD with GC CNV (r2 =0.99)” do you just mean “The SLC4A4 eQTL lead SNP is in high LD with the GC CNV (r2 204 =0.99)” The GC CNV should after all have an r2 of 1 with itself!

**AU: Thank you for pointing it out. This sentence at line 211 is now revised.**

9 – “We found strong evidence for five motifs, including transcriptional enhancer factor (TEAD) and hepatocyte nuclear factor 4 alpha (HNF4A), supporting for the presence of the active enhancer within the GC CNV (Fig 6C).” any disrupted by variants? Will their numbers change depending on copy number

**AU: These motifs were located within the GC CNV, however were not disrupted by single nucleotide polymorphisms and indels. In theory, a GC CNV carrier animal would have multiple copies of these transcription factor binding motifs.**

**Comments from reviewer 3**

The manuscript presented by Lee and collaborators reports a CNV spanning exon 14 of the group-specific component gene (GC) associated with resistance to clinical mastitis in Dutch Holstein Frisian cattle. The study is extraordinarily rich and dense, and combined multiple data sources to infer that the mentioned CNV affects an enhancer leading to increased expression of the canonical GC transcript, potentially reducing circulating vitamin D in carrier cows. Given the importance of the vitamin D pathway to immunity, and its previously hypothesized role in clinical mastitis, the results seem robust and sound. Apart from disagreeing with the use of the term “Vitamin D deficiency” in the absence of a detailed clinical and laboratorial characterization, I have only minor revisions to suggest which are listed below.

**AU: We thank the reviewer for these positive comments on the manuscript. We admit that the scope of the current study had not included clinical characterization. Thus, we revised a sentence at line number 39-40 as following:**

“We speculate that upregulation of GC leads to a large amount of vitamin D binding protein, which in turn, reduces biologically available vitamin D, **leading to** lower CM resistance.”

1 – The authors report that all analyses were performed according to the UMDv3.1 bovine assembly. Since ARS-UCDv1.2 has become the standard in the cattle community, the authors should provide the equivalent coordinates on the newer assembly. Instead of redoing part of the work, I would recommend the authors to simply report a liftOver analysis of at least the most relevant coordinates within the manuscript (ex. QTL region, CNV region, putative causal variant, and gene location). This could be done either within the text or as an additional tab in the existing supplementary file.

**AU: Thank you for your constructive feedback. We added supplementary table 1, which lists important SNPs (e.g. GWAS lead SNPs) of the current and previous studies in both UMD3.1 and ARS-UCD1.2 coordinates. We referred to this supplementary table in our manuscript as follow in line number 83:**

**“(See S1 Table for SNP positions in ARS-UCD1.2).”**

2 – Given the previous evidence from the literature supporting the QTL position in multiple populations, the GWAS analysis presented in this manuscript seems adequate and robust. However, for the sake of clarity, the authors should provide more information on the phenotypes used in their association mapping. More specifically, de-regressed estimated breeding values (dEBVs) were used as pseudo-phenotypes in the GWAS analysis, but no information of the models used to obtain these dEBVs was provided. For instance, a binary response variable was used for CM. Was the analysis based on a threshold liability regression model or a generalized linear mixed model (or something else)? Which fixed and random effects were included in the model? What was the number of records and the size of the numerator relationship matrix? How were cases and controls defined? Which methods were used to fit the model and under which settings (ex. AI-REML preceded by a number of EM iterations or Gibbs sampling with x burn in, y thinning interval and z samples)? How was the distribution of the resulting reliabilities of dEBVs (at least mean, sd and some percentile, say p90)? How were the reliabilities of EBVs computed? Since reporting this for all 60 traits may be difficult and tedious, I would encourage to do at least CM, given it is the main trait in the article. These data are important for future reference (e.g., replication of the results and comparative analyses in additional populations), and to understand how reliable were the pseudo-phenotypes since no weights were used for the residuals (recommended in the analysis of dEBVs). Perhaps the authors could include one more tab in the supplementary spreadsheet with this information, or a brief paragraph in the material and methods. If these data can be found somewhere else, please include a clear reference.

**AU: The EBVs were estimated by the Dutch national evaluation by CRV B.V. (Arnhem, the Netherlands). The evaluation was done using BLUP with provided genetic parameters estimated in a linear mixed model. Included in the model for CM are: herd*year, year*month, calving age, number of registered lactation test days, number of days between 1st and final test day, heterosis effect, recombination effect, animal effect and residual. Details on the trait and the model are provided in the e-chapter from CRV. We cited CRV e-chapter and provided more information about the EBVs in materials and methods section at line 386-389 as following:**

“**CM resistance was recorded as a binary trait, depending on the disease status registration done by farmers and on somatic cell count level of routine milk recording samples [30].** The estimated breeding values (EBVs**; obtained based on BLUP as published in the national evaluation by cooperation CRV [30])** were de-regressed to correct for the contribution of family members and de-regressed EBVs were used as phenotypes in an association analysis.”

**De-regressed proofs were received for the bulls together with effective daughter contributions (EDCs). The EDC was ranging between 25 and 971, with an average of 204 and a standard deviation of 217. We assumed homogeneous residuals in the GWAS models. This information is now incorporated in the material and methods section at line 391.**

3 – In tab 1 of the supplementary file, please indicate the direction of the effect (which allele is being counted, A1 or A2?) and which allele frequency is reported (A1 or A2?). It seems a standard GCTA output, but it is better to confirm.

**AU: Supplementary table 2 shows a standard GCTA output. This table shows the allele frequency and the estimated effect size of the A1 allele (6^th^ and 7^th^ columns, respectively). We added a footnote in supplementary table 2 and 4 to clarify this.**

4 – In the IGV screenshots of Figure S1 I miss some description regarding the unmatching sequences observed in reads located in the flanks of the CNV (chimeric reads?). They indicate that the structural arrangement may not be perfect tandem duplications, and small sequence gains/losses likely occurred between successive copies (perhaps in a heterogenous way between CN4, CN5 and CN6). A quick alignment of these sequences within the segment could provide some insight, especially on the potential role of the MIR and CACAT microhomology as “guides” or anchor/pivot points to the duplications.

**AU: Thank you for your critical comments on the structural arrangement of the GC CNV. To avoid repetition of text, please find our answers to the first question made by the reviewer 2 and see our new Figure S1 (panels F-H; new legend added at line number: 925-937).**

5 – In the discussion section the authors argue that SNP-based GWAS may miss relevant signals linked to structural variants, since these variants might be poorly tagged by SNPs because of their bi-allelic nature and of the possibility of genotyping errors caused by allelic imbalance within complex structural arrangements. They further suggest that exploratory analyses for the presence of CNV may be beneficial, especially with whole-genome sequence data. Wouldn’t it also be relevant to include a small discussion (1-2 phrases) on the possibility of using phased haplotypes as opposed to single markers to improve tagging? The EHH results presented here clearly suggest that haplotype data may be also beneficial (even with SNP Chip sparsity given the extended haplotype), and perhaps allow for prediction of causal variant carrier status in the discovery data set. This could be helpful as an additional tool to narrow down the candidate variants.

**AU: Thank you for the constructive feedback. We agree that haplotype-based approaches have been invaluable in discovery of trait-associated SVs, as with the GC CNV. As suggested, we added a sentence remarking the merit of haplotype-based approaches and cited some remarkable examples where haplotype-based approaches assisted discovery of trait-associated SVs including:**

1) **A structural variant in the 5'-flanking region of the *TWIST2* gene affects melanocyte development in belted cattle (Mishra *et al.,* 2017)**
2) **A 660-Kb Deletion with Antagonistic Effects on Fertility and Milk Production Segregates at High Frequency in Nordic Red Cattle: Additional Evidence for the Common Occurrence of Balancing Selection in Livestock (Kadri *et al.,* 2014)**

**The suggested sentence is incorporated at line 265-267 and it reads like:**

“**Note that the EHH analysis could clearly discern the GC CNV carrying haplotype (Fig 4B), showing the merit of a haplotype-based approach in delineating trait-associated structural variations, as demonstrated in earlier examples [48–51]. Also,** an exploratory check for presence of CNVs might be beneficial for fine-mapping studies.”

6 – I would include the following reference: Lippolis et al. (2011) 10.1371/journal.pone.0025479. The authors found that infusion of 25-Hydroxyvitamin D3 into the mammary gland of Holstein cows reduced the severity of experimental infection by Streptococcus uberis. Sample size was small (n = 10) but it is worth mentioning to further strengthen the authors’ hypothesis on the importance of the CNV in the etiopathogenesis of clinical mastitis in Holstein cows.

**AU: Thank you for nice suggestion. The literature is now cited in our introduction at line 65.**
